# Serum Estradiol and Testosterone Levels in Kidney Stones Disease with and without Calcium Oxalate Components in Naturally Postmenopausal Women

**DOI:** 10.1371/journal.pone.0075513

**Published:** 2013-09-23

**Authors:** Zhijian Zhao, Zanlin Mai, Lili Ou, Xiaolu Duan, Guohua Zeng

**Affiliations:** 1 Department of Urology, Minimally Invasive Surgery Center, the First Affiliated Hospital of Guangzhou Medical University, Guangzhou, China; 2 Guangdong Key Laboratory of Urology, Guangzhou, China; University of Catania, Italy

## Abstract

**Objective:**

Epidemiological data reveal that the overall risk for kidney stones disease is lower for women compared to age-matched men. However, the beneficial effect for the female sex is lost upon menopause, a time corresponding to the onset of fall in estrogen levels. The aim of this study was to describe the serum estradiol (E2) and testosterone (T) characteristics of naturally postmenopausal women with kidney stones.

**Methods:**

113 naturally postmenopausal women with newly diagnosed kidney stones (aged 57.4±4.98 years) and 84 age frequency matched stone-free controls (56.9±4.56 years) were validly recruited in the case-control study. The odds ratios (ORs) for the associations between sex hormones and kidney stones were estimated with logistic regression models, adjusting for demographic data and medical history. Patients were also stratified analyzed according to stone components (calcium oxalate stones [COS]; non-calcium oxalate stones [NCOS]).

**Results:**

Serum E2 (21.1 vs. 31.1 pg/ml) was significantly lower in kidney stones patients compared to controls. Post-hoc analysis demonstrated that this effect was driven by COS patients (p<0.001). According to tertiles of the E2 levels, a significant higher frequency of COS was seen in the lowest E2 group (p <0.001). Multiple logistic regression analysis identified E2 level as a strong factor that was independently associated with the risk for COS (per 1 SD increase, OR=0.951, 95% confidence interval [CI] = 0.919-0.985; highest: lowest tertile, OR=0.214, 95%CI = 0.069-0.665). However, serum T levels did not significantly differ among the groups.

**Conclusions:**

Naturally postmenopausal women with higher remaining estradiol levels appear less likely to suffer from kidney calcium oxalate stones. However, no correlation was found between serum T level and kidney stones. These findings support the hypothesis that higher postmenopausal endogenous estrogens may protect against kidney stones with ageing.

## Introduction

There is persuasive evidence that kidney stone formation is modulated by sex hormones in human. For example, in women with calculi a second peak in the incidence of stone formation has been reported to occur during the sixth decade of life, a time that corresponds to the onset of menopause with a fall of estrogen levels [1,2]. Epidemiological data reveal that the overall risk for kidney stones disease is lower for women compared to age-matched men [1-3]. With advancing age the hospitalization rate for urolithiasis in women decreases, attaining a value at ages greater than 70 years that is half that of the peak [4]. However, in men a single peak onset of stone disease occurs in the fourth to fifth decade, although the hospitalization rate for stones also tends to wane with advancing age [1,2,4]. Each of these observations supports a role for sex hormones in lithogenesis.

Androgens and estrogens have long been suspected of being etiologically important in the formation of calcium oxalate stones. In general, androgens appear a promotion action and estrogens appear a inhibition action on kidney stone formation, which were reported in experimental animal studies [5-8]. However, the studies were limited to calcium oxalate stones in rats. Therefore, it is great important to know which chemical component stones are regulated via the impact of androgens and estrogens. In the other hand, despite the potential pathogenetic role of sex steroids on stone formation, the clinical relevance of these observations in human subjects remains to be further elucidated. To our knowledge only 2 studies were focused on the effect of serum androgens on risk of urinary stones in males [9,10]. Peripheral conversion of testosterone to estradiol via aromatase is the primary source of estrogen in postmenopausal women. This background led us to test the association between serum testosterone (T) and estradiol (E2) concentrations and the risk of kidney stones in naturally postmenopausal women.

## Materials and Methods

### Ethics statement

The study protocol was approved by the Institutional Review Board of the First Affiliated Hospital of Guangzhou Medical University in China. Written informed consent was obtained from all subjects before study participation.

### Study subjects

We conducted a hospital-based case-control study of total 113 female patients with newly diagnosed kidney stones after menopause and 84 age frequency matched stone-free female controls. All subjects were genetically Han Chinese from Guangzhou City and surrounding regions in Southern China. Female patients who were confirmed diagnosis of kidney stones based on at least two imageological modalities (e.g. NCCT or KUB or ultrasonography or intravenous urography) were consecutively recruited between between September 2012 and December 2012 at the ward of the Urology Department of our hospital, with a response rate of 96.9%. The stones-free controls were subjects who participated in healthy checkup programs in Medical Examination Center of our hospital during the same time period when the cases were recruited. Among them, controls with frequency matched to the cases by age (±3 years) were randomly selected with a response rate of 91.3%. All individuals underwent structured questionnaire and clinical measurements.

### Structured questionnaire

In-person interviews were conducted for all cases and controls by blinding-trained interviewers using a structured questionnaire. Detailed questions were asked about age, medical history (hypertension and diabetes mellitus, estrogen use, etc.), water drink consumption (2L/d less or more), working environment temperature (28°C lower or higher), areas of life (rural or urban), age at menopause, time since menopause, body mass index (BMI). Menopause was defined as the absence of menses for a minimum of half year. Height and weight were measured with the subject in standing position wearing indoor clothes and no shoes. Body mass index (BMI) was calculated as the weight in kilograms divided by the square of the height in meters. Cases and controls were also asked whether a doctor had ever informed that they had specific stone-related diseases reported in EUA guideline (such as primary hyperparathyroidism, nephrocalcinosis, gastrointestinal diseases, bariatric surgery, primary hyperoxaluria, cystinuria, renal tubular acidosis, and anatomical abnormalities associated with stone formation, etc.).

### Inclusion criteria

All participants were female who had experienced a natural menopause. The ages ranged 50-70 years. All the women recruited should have an intact uterus and at least one intact ovary, have no use of sex steroids after the reported date of last menstruation, and have no possible endocrinopathies chronic diseases evaluated by clinically physical examination or structured questionnaire. All the subjects were also excluded if they suffered from some specific causing stone-related diseases. Only patients with kidney stones diagnosed after menopause were included in the case group.

### Stone composition analysis

Stone sample was obtained in each patient at the end of the surgery treatment (i.e., percutaneous nephrolithotomy or retrograde intrarenal surgery). Stones were analyzed with the Vector 22 Fourier transformation infrared spectrometer (Bruker, Karlsruhe, Germany) according to the manufacturer protocol. The relative proportions of the various stone components were quantified by analysis of a global powder of the sample. Calculi were classified on the basis of their main component (i.e., accounting for at least half of the stone content). Then patients with kidney stones were divided into two subgroups according to stone components (calcium oxalate stones [COS]; non-calcium oxalate stones [NCOS]). Calcium oxalate mono- and dehydrate were grouped into the single category of calcium oxalate stones (COS), and other component forms of stones were grouped into the category of non-calcium oxalate stones (NCOS).

### Blood sample measurements

Venous blood samples were collected in a tube containing 2% heparin in the morning between 8 and 10 a.m. after an overnight fast. Serum and plasma were separated and frozen at -70°C. Serum concentrations of estradiol (E2) and testosterone (T) were measured using chemiluminescence immunoassay (CLIA) on the Immulite Beckman DXI800 (Beckman Coulter, USA). The limits of detection and the intra- and inter-assay coefficients of variation, respectively, were 2 pg/ml, 2% and 5% for E2; 10 pg/ml, 2% and 4% for T. E2 and T levels below the assay limits were assigned the value of the assay limits. Thirteen women had E2 levels below the limits of the assay. 125 cases and 95 controls experienced the sex hormone detection in this study. However, of these, eight patients and eight controls were excluded because their high E2 levels (> 100 pg/ml) suggested their unreported estrogen use, while a further four and three were excluded because of total testosterone levels outside the normal physiological range (> 0.8 ng/ml). Therefore, the remaining 113 patients and 84 controls was the final cohort of this report.

### Statistical analysis

Statistical analyses were performed using SPSS for Windows (version 13·0). The data were presented as means with standard deviations and range for continuous variables, or numbers and percentages for categorical variables. Comparisons of dichotomous risk factors between cases and controls were conducted using Chi-square test; comparisons of means were conducted using Wilcoxon signed rank test. Differences were considered significant at p<0.05. Post-hoc analyses (E2 and T levels of COS versus controls, COS versus NCOS, NCOS versus controls) were corrected for multiple testing. The association between sex hormone levels and kidney stones, measured by the odds ratio (OR) and its corresponding 95% confidence interval (CI), was estimated using unconditional logistic regression, without or with adjustment for demographic data(age, BMI, at menopause, time since menopause, etc.) and medical history (i.e., hypertension and diabetes mellitus). The data were further stratified by stone composition to evaluate the stratum variable-related ORs. Spearman’s correlation was performed by using estradiol (E2) and testosterone (T) as dependent variables in all 197 subjects. Hormonal data were modeled as continuous variables and also stratified in tertiles to explore whether non-linear or threshold effects were present. Distributions of characteristics of total subjects were expressed according to the tertiles of E2level.

## Results

### Demographic variables and serum E2 and T levels in patients and controls

Table 1 provides an overview of demographic, clinical, and biochemical data of the groups. There were 113 patients with newly diagnosed kidney stones and 84 age frequency matched stone-free controls. There was no significant difference in age, time since menopause and the presence of hypertension and diabetes (p=0.491, 0.132, 0.313, 0.424, respectively) in two groups. However, patients had younger age at menopause (p=0.001), lower BMI (p=0.004), less water drink consumption (p=0.049), higher working environment temperature (p=0.046) and a rural area of life (p=0.018) compared with controls.

**Table 1:** Demographic, clinical, and biochemical data.

Variables	Controls	Kidney stones		All	p (Control vs. All)
		COS	NCOS		
N	84	74	39	113	-
Mean±SD(range) age(yrs)	56.9±4.56 (49-68)	57.1±5.0 (48-67)	57.9±4.96 (49-69)	57.4±4.98 (48-69)	0.491
Mean±SD(range) AAM(yrs)	49.9±1.28 (46-52)	49.3±1.04 (47-52)*	49.7±0.89 (48-51)#	49.4±1.01 (47-52)	0.001
Mean±SD(range)TSM (yrs)	6.9±4.78 (1-17)	7.8±5.12 (1-18)	8.2±5.02 (1-19)	8±5.08 (1-19)	0.132
Mean±SD(range)BMI(kg/m2)	24.5±3.16 (15.8-32.5)	22.4±2.45 (17.6-28.7)*	24.9±3.33 (17.3-31.2)#	23.2±3.01 (17.3-31.2)	0.004
WDC(<2L/d)	27 (32.1%)	34 (45.9%)	18 (46.2%)	52 (46%)	0.049
WET(>28°C)	13 (15.5%)	20 (27%)	11 (28.2%)	31 (27.4%)	0.046
HTN (%)	29 (34.5%)	30 (40.5%)	17 (43.6%)	47 (41.6%)	0.313
DM (%)	12 (14.3%)	14 (18.9%)	7 (17.9%)	21 (18.6%)	0.424
RA	31 (36.9%)	42 (56.8%)*	19 (48.7%)	61 (54%)	0.018
Mean±SD (range) T (ng/ml)	0.26±0.14 (0.01-0.68)	0.26±0.17 (0.01-0.84)	0.28±0.22 (0.01-0.94)	0.27±0.19 (0.01-0.79)	0.786
Mean±SD (range) E2 (pg/mL)	31.1±16.64 (2-94)	18.3±11.94 (2-78)*	26.5±14.88 (2-60)#	21.1±13.54 (2-78)	<0.001

SD, standard deviation; COS, calcium oxalate stones; NCOS, non-calcium oxalate stones; AAM, age at menopause; TSM, time since menopause ; BMI, body mass index; WDC, water drink consumption; WET, working environment temperature; HTN, hypertension; DM, diabetes mellitus; RA, rural area of life; T, testosterone; E2, estradiol. P-values were determined using the Wilcoxon rank sum test, or the chi-square tests between all patients and controls subjects. Post-hoc analyses were performed for comparison of variables between the following cohorts: COS, NCOS, controls, COS versus NCOS, COS versus controls, and NCOS versus controls, with p-values<0.05 considered significant."* "indicate group is significantly from the control."#" indicate group is significantly from the COS.

Kidney stones patients had significantly lower serum E2 (21.1±13.54 vs. 31.1±16.64 pg/ml, p<0.001) than controls, while serum T2 (0.27±0.19 vs. 0.26±0.14 ng/ml, p=0.786) was observed no difference in both groups. Stone composition analysis identified 74 COS and 39 NCOS in the 113 patients. The post-hoc analysis demonstrated that, within the kidney stones group, COS patients accounted for this effect (18.3±11.94 vs. controls 31.1±16.64 pg/ml, p<0.001; r=0.466, p<0.001). Serum E2 levels tended to be lower in COS patients than in NCOS patients (18.3±11.94 vs. 26.5±14.88 pg/ml, p=0.002), but was not significantly different between NCOS patients and controls (p=0.142).

Spearman correlations using estradiol (E2) and testosterone (T) as a dependent variables are shown in Table 2. E2 level was not associated with age (p=0.137), age at menopause (p=0.154), time since menopause (p=0.098), but was significantly related to T level (r=0.204, p=0.002), BMI (r=0.583, p<0.001) and stones components (r=0.365, p<0.001). Using partial correlation analysis, controlling for BMI, positive associations between E2 levels and stones components (r=0.241, p=0.001) remained significant across the combined cohort, whilst association with T level was no longer significant. However, T level was not associated with stones components in spearman correlations (p>0.05).

**Table 2:** Spearman’s correlation using estradiol (E2) and testosterone (T) as dependent variables in all 197 subjects.

	Spearman’s rank	T	BMI	Age	AAM	TSM	HTN	DM	WDC	WET	RA	Stones components (control/COS/NCOS)
	correlation											
E2	r	0.204	0.583	-0.078	0.073	-0.093	0.073	0.098	-0.115	0.077	0.091	0.365
	p	0.002	<0.001	0.137	0.154	0.098	0.152	0.085	0.054	0.141	0.102	<0.001
T	r	-	0.164	0.078	-0.070	0.091	0.100	0.062	-0.102	-0.110	-0.001	-0.006
	p	-	0.011	0.139	0.165	0.101	0.081	0.193	0.077	0.061	0.496	0.465

### Associations of sex steroids with kidney stones in postmenopausal women

Logistic regression models examining the association of sex hormones as continuous variables with the presence of kidney stones are shown in Table 3. After adjusting for BMI, water drink, age at menopause, area of life, working environment temperature, the increased E2 was associated with decreased risk of kidney stones (per 1 SD increase, odds ratio [OR]: 0.952, 95% confidence interval [CI]: 0.924-0.980, p=0.001), but T was not (OR: 2.13, 95%CI: 0.290-15.648, p=0.458). Moreover, the effect of increased E2 was more profound in reducing the kidney COS risk (per 1 SD increase, OR: 0.951,95% CI: 0.919-0.985; p=0.005). In addition, in the fully- adjusted model, age at menopause and water drink consumption were independent risk factors of kidney stones (each p<0.05).

**Table 3:** Determinants of kidney stones (KS) and calcium oxalate stones (COS) for all 197 subjects, respectively.

Variable		Univariate		Multivariate	
		OR (95% CI); p-value		OR (95% CI); p-value	
AAM(yrs)	KS	0.647 (0.497-0.841); p=0.001		0.641 (0.477-0.861); p=0.003	
	COS	0.611 (0.464-0.804); p<0.001		0.579 (0.421-0.797); p=0.001	
BMI (kg/m2)	KS	0.870 (0.791-0.957); p=0.004		0.978 (0.862-1.109); p=0.729	
	COS	0.763 (0.682-0.855); p<0.001		0.841 (0.726-974); p=0.021	
WDC(<2L/d)	KS	1.80 (0.999-3.242); p=0.05		2.427 (1.219-4.830); p=0.012	
	COS	1.473 (0.820-2.648); p=0.195		2.098 (1.026-4.292); p=0.042	
RA	KS	2.006 (1.126-3.572); p=0.018		1.645 (0.847-3.195); p=0.142	
	COS	1.916 (1.069-3.436); p=0.029		1.474 (0.742-2.982); p=0.269	
E2 (pg/mL)	KS	0.955 (0.934-0.976); p<0.001		0.952 (0.924-0.980); p=0.001	
	COS	0.936 (0.911-0.963); p<0.001		0.951 (0.919-0.985); p=0.005	
WET>28°C	KS	2.065 (1.004-4.248); p=0.049		1.466 (0.645-3.337); p=0.361	
	COS	1.528 (0.744-3.015); p=0.222		1.300 (0.5822-2.906); p=0.522	
Total T (ng/ml)	KS	1.258 (0.242-6.540); p=0.785		2.13 (0.29-15.65); p=0.458	
	COS	0.960 (0.169-4.865); p=0.908		2.573 (0.327-20.253); p=0.369	

OR = odds ratio; CI = confidence intervals.

Hormonal data were modeled as continuous variables, and also stratified in tertiles to explore whether non-linear or threshold effects were present (Table 4 and Table 5). A significant decrease in the frequency of kidney stones was seen with increased tertiles of E2 concentration (p <0.001). As expect, a same trend was observed in frequency of COS patients (p<0.001), while no difference was seen in the frequency of kidney NCOS patients (p=0.705). According to the tertiles of E2 levels, in the fully-adjusted model, women with E2 in the highest tertiles had significantly decreased kidney stones risk compared with women with the lowest tertile (OR: 0.141,95% CI: 0.062-0.324, p<0.001). The unadjusted and adjusted ORs for COS formation also tended to be significant rarer in the highest tertile of E2 (OR: 0.121, 95%CI: 0.051-0.287, p<0.001 and OR: 0.214, 95%CI: 0.069-0.665, p=0.008, respectively) than in the lowest one.

**Table 4:** Characteristics of the study population (n =197) per tertile of estradiol.

		Lowest tertile	Middle tertile	Highest tertile	
Variables	Study population	2-17pg/ml	18-29 pg/ml	31-94 pg/ml	p value
N	197	64	73	60	—
Mean(SD) E2 (pg/mL)	25.4 (15.70)	10.4 (4.82)^a^	23.5 (3.57)^b^	43.7 (13.8)^c^	<0.001
Kidney stones (%)	113	50 (78.1%)^a^	40 (54.8%)^b^	23(38.3.0%)^c^	<0.001
NCOS (%)	39	12 (18.7%)	13 (17.8%)	14 (23.3%)	0.705
COS (%)	74	38 (59.4%)^a^	27 (37.0%)^b^	9 (15%)^c^	<0.001

^a, b, c^ Different superscripts indicate significant differences among groups, p<0.05.

**Table 5:** Odds ratios (OR) and 95% confidence intervals (CIs) for kidney stones (KS) and calcium oxalate stones (COS) according to tertiles of E2 levels.

			Lowest tertile ^a^	Middle tertile	Highest tertile	p for trend
			OR (95% CIs); p-value			
serum E2(pg/ml)			2-17	18-29	31-94	
Unadjusted:		KS	1	0.452 (0.244-0.910); p=0.026	0.162 (0.073-0.358); p<0.001	<0.001
		COS	1	0.402 (0.202-0.800); p=0.009	0.121 (0.051-0.287); p<0.001	<0.001
Adjusted factors^b^:		KS	1	0.431 (0.227-0.896); p=0.024	0.141 (0.062-0.324); p<0.001	<0.001
		COS	1	0.518 (0.218-1.228); p=0.135	0.214 (0.069-0.665); p=0.008	P=0.029

^a^ Reference OR (1.00) is the lowest tertile of E2 level for kidney stones (KS) and calcium oxalate stones (COS). ^b^ Adjusted factors=Age +AAM + TSM + BMI + WDC + WET +HTN + DM +RA +T

## Discussion

In our case-control study first investigating serum E2 and T values of postmenopausal women with kidney stones, lower serum E2 levels were significantly associated with the odds for increased risk of kidney stones. The effect was mainly driven by COS patients. However, no correlation was found between serum T level and kidney stones in this cohort.

We found E2 levels were not associated with age, age at menopause, time since menopause but positively associated with BMI. These findings are consistent with previously reported postmenopausal evolution of sex steroid serum concentrations, with intraindividual E2 levels remaining relatively stable from 1 year after postmenopausal decline [11]. The principal source of oestrogens in postmenopausal women is peripheral aromatization of androstenedione to oestrone in adipose tissue and skin [12]. Postmenopausal estrogen production is thus primarily influenced by body weight but not by age [11].

Interestingly, in our population serum E2 levels increased with BMI, and frequency of kidney stones decreased with higher serum E2 levels, but higher BMI did not protect against kidney stones. Fat mass or higher BMI was not related to stones, which suggests the importance of other sources of estradiol production than adipose tissue in postmenopausal women. In fact, extragonadal oestrogen biosynthesis occurs in a number of sites, including adipose tissue, bone, various sites of the brain, and vascular endothelial and smooth muscle cells [12]. On the other hand, obesity and weight gain increase the risk of kidney stone formation [13,14]. However, the prevalence of stone disease may not increase in concert with increasingly obese BMI values when the BMI values are below the threshold of 30kg/m^2^ [28], which is corresponding to our results. Extremely obesity is associated with insulin resistance and compensatory hyperinsulinemia, metabolic derangements, and urine chemistry that may lead to the formation of calcium-containing kidney stones [13-15].

Serum T levels were not significantly different between patients and controls in our study. However, Li et al. [9] and Justin et al. [10] reported that male stone formers had higher serum total testosterone levels compared with a similar cohort without stones. These results were consistent with the promotion action of testosterone for kidney stones in several animal models. Our inconsistent findings may have been due to the gender and age diversities. Levels of adrenal androgens also decline with increasing age in both men and women [16]. Nordin et al. [17] have reported the negative impact of declining dehydroepiandrosterone (DHEA) on vertebral bone mineral density and intestinal calcium absorption in osteoporotic postmenopausal women.

Estrogen has been shown to inhibit bone resorption and enhance renal tubular calcium re-absorption [18,19]. Previous studies of stone forming patients also showed that men did excrete more calcium and oxalate (two important promoters of lithogenesis) and less citrate (an important inhibitor of lithogenesis) than women [20-22]. Several changes occur in women as they approach menopause. The values of urinary calcium in males and females converged at the time of menopause (about age 50 years) [23]. It has been proposed that the excretion of urinary citrate varies with serum estrogen during the menstrual cycle [20]. These studies are compatible with the hypothesis that menopause is associated with loss of the hypocalciuria and hypercitraturia effect of estrogen and it may explain the second peak age of onset for nephrolithiasis in women [1,2]. Menopausal estrogen deficiency in women is associated with reduced calcium absorption and increased calcium oxalate saturation, which is reversed by estrogen replacement [18,19,23,24]. These findings support a connection of serum estrogen status with the propensity for calcium oxalate nephrolithiasis.

In experimental animal studies, Lee [5], Lguchi [6], Yoshioka [7] and Fan [8] also found extensive crystal deposition in intact male rats and testosterone -administered males and females, whereas relatively few crystal deposits were observed in intact females, castrated females, castrated males, and estradiol -administered males. In addition, castration of male rats dramatically decreased the incidence of renal stones. Their findings showed that testosterone is a promoter and estradiol an inhibitor of crystal deposition [5-8]. Some researchers focused on the impact of the sex hormonal on the active renal calcium re-absorption. These studies shows that E2 and T are associated with coordinated changes in expression of many active renal calcium transports (i.e., TRPV5, calbindin-D28K, plasma membrane Ca^2+^-ATPase, and Na+/Ca^2+^-exchanger) involved in distal renal tubule calcium re-absorption [25-27].

Serum E2 levels were not significantly different between NCOS patients and controls. This result may motivate to future studies focusing on putative interaction pathways between estradiol and kidney calcium oxalate stones only. The difference of E2 levels between COS and NCOS patients may be due to sample size, but also to the fact that testosterone promotes and estradiol inhibits the liver glycolate oxidase, which converts glycine to oxalate, affecting urinary oxalate excretion [28]. Together with a significant negative correlation between urinary calcium and plasma estradiol levels, the clinical relevance of these observations in human subjects was first reported in our study and can be arguably understand.

Although this study is one of the few to test the association of sex hormones and kidney stones and is the first focusing on populations of female. However, we did not evaluate serum sex hormone in pre-menopause female since it was difficult to exclude the effect of physiological fluctuation due to the menstrual cycle. Our study also had other limitations. Sample size was not large and hormone levels were only measured at a single time point. Finally, our study does not contain information on 24-hour urinary composition, thus limiting the conclusions that can be made about the role of sex hormones on urinary composition, which was an important factor of kidney stone formation.

In conclusion, naturally postmenopausal women with higher ‘remaining’ estradiol levels appear less likely to suffer from kidney calcium oxalate stones. This effect seems independent of age at menopause, time since menopause but correlated with body mass index. However, no correlation was found between serum T level and kidney stones regardless the stone components in this age group. Further studies are needed to examine underlying mechanisms and evaluate therapeutic options in this group of women.
